# Relations between Psychological Needs Satisfaction, Motivation, and Self-Regulated Learning Strategies in Medical Residents: A cross-sectional Study

**DOI:** 10.15694/mep.2018.0000087.1

**Published:** 2018-04-18

**Authors:** Fareeda Mukhtar, Krista Muis, Michelle Elizov

**Affiliations:** 1 McGill University

**Keywords:** Motivation, Self-regulated learning, Residents education, Basic psychological needs

## Abstract

This article was migrated. The article was marked as recommended.

Residents in the medical field work to fulfil their clinical duties and study to pass exams at the same time. Thus, they need to continuously learn and acquire knowledge in a self-regulated manner that accommodates their busy work schedule. The importance of self-regulated learning (SRL) and its relation to motivation is widely recognized in educational literature, yet it is still not sufficiently explored in medical education literature. The relationship between self-regulated learning (SRL) and motivation has not been sufficiently explored among medical residents. A total of 160 residents from different medical departments at McGill University were asked to complete a questionnaire about their psychological needs satisfaction, motivation to learn, and use of SRL strategies. Our results showed that residents who are more intrinsically motivated reported more utilization of SRL strategies. Results are discussed in terms of their impact on medical education practice as well as their theoretical implications.

## Introduction

Medical professionals are required to remain current with the constantly expanding medical knowledge to maintain high-quality health care. It is well recognized that they need to learn continuously as part of their daily practice ( Van de Wiel, Van den Bossche, Janssen, & Jossberger, 2011). That said, the knowledge of medical professionals has been shown to decline with time, potentially resulting in lower quality of care( Choudhry, Fletcher, & Soumerai, 2005). One of the main explanations of this phenomenon is the lack of updated knowledge and skill through self-regulated learning (SRL)( Choudhry et al., 2005). Paul R Pintrich (2000) defines SRL as “an active constructive process whereby learners set goals for their learning and then attempt to monitor, regulate, and control their cognition, motivation and behavior, guided and constrained by their goals and the contextual features in the environment” (p. 453). SRL does not develop automatically, thus, the importance of incorporating SRL during medical training is now widely recognized ( Brydges & Butler, 2012; Winne, 2005). Unfortunately, there is little published evidence that medical schools and post-graduate institutions are successfully helping students and residents become effective self-regulated learners ( Lucieer, Jonker, Visscher, Rikers, & Themmen, 2016; White, Gruppen, & Fantone, 2010).

Residents represent a special group of health professionals. Since residents are transitioning from being medical students into becoming independent practitioners in various medical and surgical specialties, they carry a complex mixture of responsibilities. During this period, residents are responsible for actively contributing to the solution of patients’ healthcare problems. At the same time, they are studying continuously to increase their knowledge, pass exams, and perfect their skills. Residents are challenged with time restrictions related to trying to balance their personal life, professional duties, and study ( Duffy, 2008; Lacasse, Lee, Ghavam-Rassoul, & Batty, 2009). The heavy workload and the continuous learning responsibility required from residents reflect the complexity of their work/learning environment and the concurrent necessity for optimizing their use of SRL strategies ( Ten Cate, Kusurkar, & Williams, 2011).

SRL is highly context-dependent; therefore, without setting the optimal conditions for residents to utilize SRL strategies, residents are not readily becoming efficient self-regulated learners ( Van de Wiel et al., 2011; White et al., 2010; Wyatt & Sullivan, 2005). A key element to enhance SRL is the achievement of high levels of intrinsic motivation ( Reeve, Ryan, Deci, & Jang, 2007). Intrinsic motivation is defined as a person doing an activity for its inherent satisfaction rather than for external consequences ( Deci & Ryan, 2000). In contrast to the extrinsic motivation, which is doing an activity to attain an external outcome, such as rewards, or to avoid a negative outcome, such as punishment ( Deci & Ryan, 2000).

The relationship between intrinsic motivation and SRL has been demonstrated in multiple empirical studies done in classroom environments ( Reeve et al., 2007; Williams & Deci, 1996). A few studies have examined intrinsic motivation and SRL in medical residents. Van de Wiel et al. (2011) described the use of SRL among residents as disappointing and sub-optimal. Besides their busy schedules, a lack of intrinsic motivation has been described as a primary reason for these results ( Lacasse et al., 2009; Van de Wiel et al., 2011). R. A. Kusurkar, Croiset, and Ten Cate (2011) have suggested different strategies to enhance intrinsic motivation, including providing a suitable autonomy-supportive environment to augment residents’ intrinsic motivation.

Self-Determination Theory (SDT) postulates that for a person to be intrinsically motivated and have a sense of self-determination, three innate and fundamental basic psychological needs (BPN) must be satisfied, namely autonomy (i.e., sense of ownership over actions), competence (i.e., performing the task with confidence and effectiveness), and relatedness (i.e., to support and be supported by others while performing certain tasks) (Richard M. Ryan & Deci, 2002). The SDT also state that these three BPN are complementary to each other. Thus, the social context (e.g., work or academic environments) must satisfy all three psychological needs to reach the desired outcomes. SRL can be considered as a desired outcome of this theoretical framework, mediated or moderated by high levels of intrinsic motivation ( Deci & Ryan, 2000; Dysvik & Kuvaas, 2011; Gagné & Deci, 2005; Guay, Boggiano, & Vallerand, 2001). There is scant literature, to our knowledge, that directly situates self-determination theory within SRL for residents ( Sockalingam et al., 2016). Given the importance of SRL for residents, it is important to examine the extent to which medical residents use SRL strategies as well as the factors that can potentially affect the use of these strategies.

Based on theoretical and empirical considerations, we examined the relationship between the perceived levels of BPN at the work environment, the reported level of SRL strategies used by residents from various medical and surgical specialties, and the role of intrinsic motivation to mediate relations between the BPN and use of SRL strategies. In our study, we consider SRL strategies to be an outcome itself of the satisfaction of BPN.

## Methodology

We conducted a cross-sectional study on residents in various clinical specialties from McGill University Health Centre - Quebec, Canada. Upon obtaining the ethics approval in January 2014 from the Institutional Review Board (IRB) of the Faculty of Medicine at McGill University (approval numberA01-E05-14), emails were sent to each program director to attain approval to contact their residents. Residents were sent invitation emails to anonymously participate in the study by answering an online questionnaire. Hard copy versions of the questionnaire were also made available and were distributed through collaboration with the departments’ administrative assistants to increase our reach area.

Age, sex, specialty and post-graduate year (PGY) level was collected as demographic data (see Table 1). We also asked the residents to answer whether they have been previously exposed to problem based learning (PBL), as PBL courses have been associated with better utilization of SRL strategies by medical students ( Evensen, Salisbury-Glennon, & Glenn, 2001; Turan, Demirel, & Sayek, 2009).

The Questionnaire contained three validated scales to measure (BPN), motivation and use of SRL strategies. All scales were rated on a seven-point Likert scale. When necessary, we re-worded some items to appropriately reflect the residents’ environment (e.g., use residents instead of students and rotation instead of class).

The Basic Psychological Needs Scale (BPNS) was used to measure autonomy, competence, and relatedness variables. This questionnaire was based on the SDT ( Deci & Ryan, 2000). The work version consists of 21 items. For the present study, we used a shortened version of 15 items (5 items for each of autonomy, competence and relatedness), given that residents’ free time is limited and also that there are multiple other variables to be measured (i.e., intrinsic motivation, extrinsic motivation, and SRL variables).

**Figure 1.**
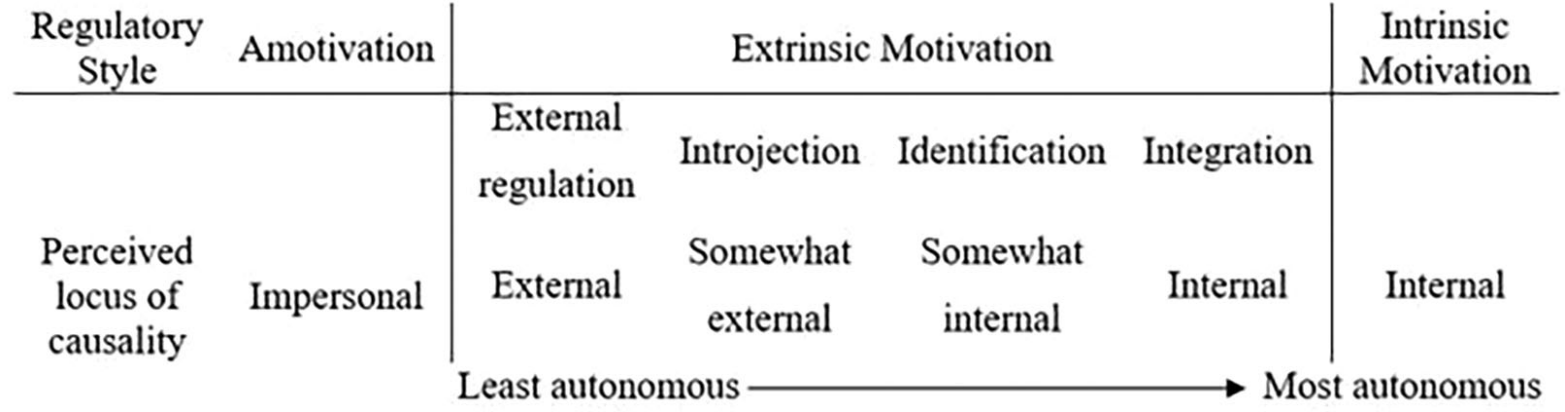
A Taxonomy of Human Motivation (adapted from Ryan & Deci, 2000)

Adapted with permission from Richard M. Ryan and Deci (2000)

The College version of the Academic Motivation Scale (AMS; (Robert J. Vallerand et al., 1992) was used to measure residents’ motivation. AMS is a validated tool to measure motivation according to SDT taxonomy ( Figure 1). AMS consists of 28 items that are grouped into intrinsic motivation and extrinsic motivation. Residents indicated to what extent each item corresponds to the reasons why they joined residency program.

The Motivated Strategies for Learning Questionnaire (MSLQ; ( Paul R. Pintrich, Smith, Garcia, & McKeachie, 1993) was used to measure residents’ self-reported learning strategies. MSLQ is a validated self-report instrument to measure students’ learning strategies. Thirty items for self-reported learning strategies were used. These items are classified into three subscales: (1) cognitive strategies (i.e., rehearsal, elaboration, organization, and critical thinking, (2) metacognitive strategies; and (3) resource management strategies (e.g., time management, effort regulation, help seeking and peer learning).

**Table 1.** General demographics

Variable	*n*	%
**Sex**		
Male	75	46.9
Female	84	52.5
Missing	1	0.6
**Age**		
23-25	27	16.8
26-30	91	56.9
31-35	37	23.1
36-38	4	2.5
Missing	1	0.6
**PGY level**		
PGY- 1	49	30.6
PGY- 2	38	23.8
PGY- 3	36	22.5
PGY- 4	21	13.1
PGY- 5	10	6.3
PGY- 6	4	2.5
Missing	2	1.3
**Department**		
Anesthesiology	13	8.1
Emergency medicine	9	5.6
Family medicine	17	10.6
General surgery	31	19.4
Internal medicine	41	25.6
Obstetrics and gynecology	16	10.0
Pediatrics	23	14.4
Radiology	10	6.3

## Results

IBM SPSS 19 was used for data analysis along with MEDIATE macro for SPSS. We first checked for the normality of each subscale of the three questionnaires (i.e., BPNS, AMS, and MSLQ). Descriptive statistics were then generated for demographics data and questionnaire scores (i.e., means, standard deviations). Cronbach’s alpha (reliability score) was calculated for each subscale in the three questionnaires ( Table 2). Pearson’s correlation coefficients between the subscales of the three questionnaires are presented in ( Table 3).

**Table 2.** Descriptive statistics and Cronbach’s α for BPNS, AMS, and MSLQ

Scale	Mean	SD	Cronbach’s *α*
Autonomy	**4.25**	**.87**	**.607**
Competence	**5.25**	**.84**	**.649**
Relatedness	**5.43**	**.83**	**.723**
Intrinsic motivation	**4.95**	**1.04**	**.902**
Extrinsic motivation	**4.68**	**.91**	**.739**
Cognitive strategies	**4.10**	**.85**	**.743**
Metacognitive strategies	**4.52**	**1.00**	**.573**
Resource management strategies	**4.07**	**.80**	**.705**

**Table 3.** Pearson’s correlation coefficients between subscales of BPNS, AMS, and MSLQ

		1	2	3	4	5	6	7	8	9
1	**Autonomy**	**1**								
2	**Competence**	**.517 ^b^**	**1**							
3	**Relatedness**	**.477 ^b^**	**.489 ^b^**	**1**						
4	**IM**	**.227 ^b^**	**.329 ^b^**	**.245 ^b^**	**1**					
5	**EM**	**.076**	**.191 ^a^**	**-.030**	**.404 ^b^**	**1**				
6	**AM**	**-.402 ^b^**	**-.538 ^b^**	**-.299 ^b^**	**-.372 ^b^**	**-.098**	**1**			
7	**CG**	**.036**	**.173 ^a^**	**.008**	**.339 ^b^**	**.147**	**.008**	**1**		
8	**MC**	**.160 ^a^**	**.283 ^b^**	**.120**	**.331 ^b^**	**.033**	**-.154**	**.665 ^b^**	**1**	
9	**RM**	**.299 ^b^**	**.403 ^b^**	**.243 ^b^**	**.359 ^b^**	**.006**	**-.21 ^b^**	**.563 ^b^**	**.606 ^b^**	**1**

Note: IM = intrinsic motivation, EM = extrinsic motivation, AM = amotivation, CG = cognitive strategies, MC = metacognitive strategies, RM = resource management strategies

^a^ Correlation is significant at .05 level

^b^ Correlation is significant at .01 level

***Demographics**.* A total of 198 residents filled out the questionnaire (117 online and 81 hard copy). Out of these, a total of 160 responses - 86 responses from the online questionnaire and 74 responses from the hard copy questionnaire - had complete answers on all 3 scales and were included in subsequent analysis. The residents who participated in the study represent all of the included departments (i.e., anesthesiology, emergency medicine, family medicine, general surgery, internal medicine, obstetrics and gynecology, pediatrics, and radiology) and their subspecialties (e.g., cardiology, endocrinology, cardiac surgery, etc.). Residents from all post-graduate year levels (PGY 1 - PGY 6) participated in the study ( table 1). Although 60.6% of residents participating in our study reported previous exposure to a problem based learning (PBL) environment, this did not seem to have any significant measurable effect on their use of SRL strategies.

***Bivariate Correlations***. The bivariate correlations presented in Table 3 show a statistically significant positive relationship between intrinsic motivation and each SRL strategy (i.e., cognitive strategies *r = .*339, *p* < .01, metacognitive strategies *r = .*331, *p* < .01, and resource management strategies *r = .*359, *p* < .01). In contrast to intrinsic motivation, extrinsic motivation did not show any significant bivariate correlation with any of the SRL strategies.

Each of the three BPN had a statistically significant relationship with intrinsic motivation, *r = .*227, *r = .*329, *r = .*245, *p* < .01 for autonomy, competence, and relatedness, respectively. Autonomy had a significant positive correlation with metacognitive strategies *r = .*160, *p* < .05 and resource management strategies *r = .*229, *p* < .01. Competence had a significant positive relationship with the three SRL strategies; cognitive strategies *r = .*173, *p* < .05, metacognitive strategies *r = .*283, *p* < .01, and resource management strategies *r = .*403, *p* < .01). Relatedness showed a significant positive relationship only with resource management strategies *r = .*243, *p* < .01.

**Figure 2.**
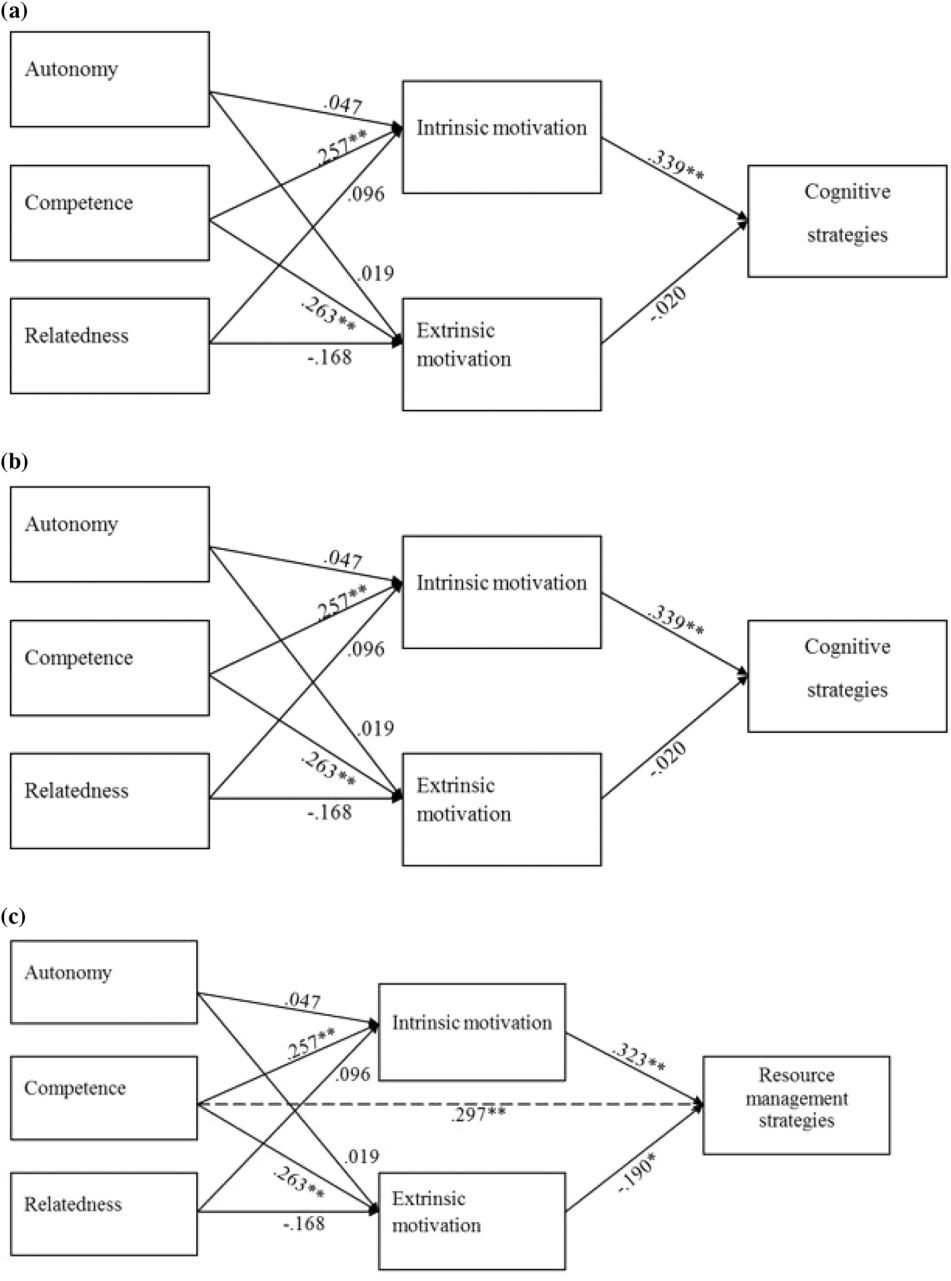
Path analysis for cognitive (2a), metacognitive (2b) and resource management (2c) SRL strategies

***Path Analysis***.The MEDIATE macro (Preacher & Hayes, 2008) for IBM SPSS 19 was used to examine the direct and indirect relationships between BPN, motivation, and SRL strategies according the diagram illustrated in Figure 2. This model is used for each SRL strategy separately. We first examined the relationship between BPN, intrinsic motivation (IM), and extrinsic motivation (EM) as predictors of cognitive SRL strategies, as illustrated in Figure 2a. The model was statistically significant *F* (5,154) = 4.85, *p* < .01, ( *R ^2^* = 13.62%). Competence had a significant direct relationship with intrinsic motivation *B* = .257, *t* (158) = 2.77, *p* <.01, and extrinsic motivation *B* = .263, *t* (158) = 2.74, *p* < .01. Cognitive strategies were significantly predicted from intrinsic motivation *B* = .339, *t* (158) = 3.92, *p* <.01. No mediation was found.

Then we examined the relationship between BPN, intrinsic motivation, and extrinsic motivation as predictors of metacognitive SRL strategies [ Figure 2b]. The model was statistically significant *F* (5,154) = 6.04, *p* < .01, ( *R ^2^* = 16.41%). Metacognitive SRL strategies were significantly predicted from competence *B* = .238, *t* (158) = 2.53, *p* < .05 and intrinsic motivation *B* = .332, *t* (158) = 3.89, *p* < .01. Competence had a significant direct relationship with intrinsic motivation *B* = .257, *t* (158) = 2.77, *p* <.01. The confidence interval of the indirect effect of competence on metacognitive SRL strategies was (.029 - .214), which indicates a complete mediation between competence and metacognitive strategies through intrinsic motivation.

Finally, we examined the relationship between BPN, intrinsic motivation, and extrinsic motivation as predictors of resource management SRL strategies ( Figure 2c). This model was statistically significant *F* (5,154) = 10.655, *p* < .01, ( *R ^2^* = 25.70%). Resource management strategies were significantly predicted from competence *B* = .297, *t* (158) = 3.35, *p* < .01, and intrinsic motivation *B* = .323, *t* (158) = 4.02, *p* < .01. Competence had a significant direct relationship with intrinsic motivation *B* = .257, *t* (158) = 2.77, *p* <.01. The confidence interval of the indirect effect of competence on metacognitive SRL strategies was (.026 - .176), which indicates a complete mediation between competence and resource management strategies through intrinsic motivation.

Path analyses for the three SRL strategies were also conducted, by controlling for demographics as covariates (i.e., age, sex, department, PGY-level, and previous exposure to PBL). None of the covariates showed a significant change in results, thus were not included in the models.

## Discussion

The process and framework of SRL has been described by multiple theorists and well-studied in the class-room environment ( Paul R Pintrich, 2000; Schunk & Zimmerman, 1998; Winne & Hadwin, 2008). The main purpose of this study was to find the relations between satisfying the three BPN, intrinsic motivation, and different SRL strategies in the residents’ more complex training and learning environment as there is a paucity of similar data in the medical education literature. We invited residents from multiple medical specialties (i.e., medical, surgical, and non-clinical) to better represent different working environments. The sample size was sufficient to have good statistical power for path analysis and was representative of all included departments and different PGY levels.

Our data is consistent with SDT; each of the three BPN showed a positive relationship with intrinsic motivation. Residents who felt more autonomous at work reported using more metacognitive and resource management SRL strategies. Residents who felt more competent at work reported using more cognitive, metacognitive, and resource management SRL strategies. On the other hand, residents who felt more relatedness to other people in the working environment reported using more resource management strategies only. Our findings are consistent with other studies which conclude that the perception of autonomy and competence for clinicians is thought to reflect positively on their SRL behaviors ( Brydges & Butler, 2012; Sandars & Cleary, 2011). Additionally, Stok-Koch, Bolhuis, and Koopmans (2007) suggest that feelings of relatedness can help residents focus on their learning process.

Results from the correlation analysis revealed that intrinsic motivation was an important predictor for the three SRL strategies; in direct contrast to extrinsic motivation. Our findings contradict Van de Wiel et al. (2011) conclusion that residents are only extrinsically motivated by their patients to provide high-quality patient care. We believe that this is not an accurate interpretation of this attitude, as health care professionals have traditionally utilized the pathologies they encounter during their duty as opportunities to learn more about the pathologies rather than merely as a search for answers to manage a specific patient ( Bethune & Brown, 2007; Fafard & Snell, 1989). In the context of the dichotic work/learn environment within which residents work, this attitude can actually be an example of residents’ utilization of metacognitive and resource management strategies during work and does not reflect an absence of an intrinsic motivation to learn.

Results from the path analysis clearly revealed the importance of residents’ self-perceived level of competence. Residents who felt satisfied in their level of competence were intrinsically motivated to learn and reported more use of SRL strategies. In fact, residents who felt more competent reported more use of metacognitive and resource management strategies consistently through all PGY levels and all departments. This is of particular interest, as residents are expected to be more competent as they progress in PGY levels as a result of their acquisition of knowledge and skill through their years of training. Hence, the reported levels of confidence would likely reflect a relative competence to what residents feel is appropriate to their level of training. This would suggest that fostering feelings of competence in residents can potentially lead to more use of SRL strategies. This is an important finding that can have multiple practical implications to be explored.

Most of the studies conducted in the medical field have focused on satisfaction of autonomy, which will lead to more autonomous motivation (i.e., intrinsic motivation) and thus increase use of SRL strategies and academic achievements ( R. Kusurkar, Ten Cate, Vos, Westers, & Croiset, 2013; Sobral, 2004). However, in our path analysis, autonomy satisfaction did not stand out as a significant driver of SRL strategies. The reason could be statistical in nature as the path analysis model tends to highlight the most significant predictors and underestimate other factors that share their variance. A noted high degree of correlation between autonomy, competence and relatedness in the bivariate analysis supports this interpretation. However, this could be due to the fact that working in a hospital environment and dealing with patients’ lives will always imply limited autonomy of the medical teams with, rightfully, continuous supervision and guidance from more experienced medical staff. This will limit the perception of autonomy in favor of ensuring patient safety. Hence, the variability of autonomy satisfaction maybe smaller than what our sample could detect.

Like autonomy, the feeling of relatedness did not show a significant effect on the use of SRL strategies in our path analysis model. The correlation between perceived competence and relatedness was high ( Table 3). This indicates that residents who felt competent at work also felt related to their surrounding environment. Therefore, competence might potentially play a role in boosting relatedness; however, we cannot verify this effect from the available study data. Previous studies have noted that satisfying one of the three psychological needs can lead to satisfying the others, which could explain the noted association ( Deci et al., 2001; Jang, Reeve, Ryan, & Kim, 2009; Ten Cate et al., 2011; Robert J Vallerand, Fortier, & Guay, 1997; Williams, McGregor, Zeldman, Freedman, & Deci, 2004).

A notable limiting factor in this study’s design was the sole reliance on self-reports for measuring SRL strategies. However, self-reporting has been recognized as a reliable method of measuring the use of different learning strategies ( Winne, Jamieson-Noel, & Muis, 2002). Additionally, self-reporting has been recognized as a reliable method of measuring the perceived satisfaction of BPN ( Ntoumanis, 2005; Richard M Ryan & Deci, 2006; Robert J Vallerand et al., 1997). Another limitation that was brought up by participants is the lack of time to read, study, and use different SRL strategies. Although, the working hours are quite similar between departments, the actual number of hours spent by resident varies according to department, workload, and number of night/weekend duties for each resident. This time factor was difficult to assess and measure in our cross-sectional study design. We believe that the time factor may represent an important covariate for SRL strategies. This issue should be examined in future longitudinal study designs.

There are several ways that this study can add to medical education literature. First, this is the first study, to our knowledge, that directly links BPN, motivation, and SRL for medical residents. Some studies focused on motivation and how it can impact the use of SRL strategies ( Artino et al., 2012; R. Kusurkar et al., 2013; Stegers‐Jager, Cohen‐Schotanus, & Themmen, 2012; Van de Wiel et al., 2011) and academic achievements ( R. Kusurkar et al., 2013; Sobral, 2004; Turan & Konan, 2012). Adding the element of BPN to this equation leads to practical implications, as residents’ perceptions of BPN can be altered by different educational strategies. Our study also contributes to the medical education literature by solidifying the idea that satisfying the need of competence for residents is an important predictor for their intrinsic motivation and use of SRL strategies. Educators can play a major role in feeding feelings of competence or incompetence in their trainees, depending on their attitudes and methods of instruction, feedback and assessment. Our data suggests that fostering the feeling of perceived competence, that is appropriately reflective of a residents’ level of training and skills, will encourage the residents to learn and work more efficiently as they become more intrinsically motivated.

## Take Home Messages

• Residents in the medical field work to fulfil their clinical duties and study to pass exams at the same time. Thus, they need to continuously learn and acquire knowledge in a self-regulated manner.
• There is little published evidence that medical schools and post-graduate institutions are successfully helping students and residents become effective self-regulated learners.
• Without setting the optimal conditions for residents to utilize their self-regulated learning (SRL) strategies, residents are not readily becoming efficient self-regulated learners.
• Our data suggests that fostering the feeling of perceived competence, that is appropriately reflective of a residents’ level of training and skills, will encourage the residents to learn and work more efficiently as they become more intrinsically motivated.

## Notes On Contributors

**Fareeda Mukhtar, M.B B.S, M.A.** Post-graduate student obtained Masters of Arts in Educational Psychology, Health Profession stream, McGill University, Canada. Currently at the Multidisciplinary Simulation center, Mayo Clinic, Rochester MN, USA.

**Krista Muis, Ph.D,** Associate Professor and Canada Research Chair Tier II in Epistemic Cognition and Self-Regulated Learning, Department of Educational and Counselling Psychology, Faculty of Education, McGill University.

**Michelle Elizov, MD, MHPE, FRCPC,** Associate Professor of Medicine at McGill University and an attending physician at the Jewish General Hospital. She is the Director of University and Medical Education, CIUSSS Centre Ouest de l’Ile de Montreal.
