# Supplementary material for: Relations between Psychological Needs Satisfaction, Motivation, and Self-Regulated Learning Strategies in Medical Residents: A cross-sectional Study
Source: MedEdPublish (2016). 2018 Apr 18;7:87. [Version 1] doi: 10.15694/mep.2018.0000087.1 (PMC10712002; doi:10.15694/mep.2018.0000087.1)
Supplement: Peer review history

# Reviewer report: Reviewer response for version 1

**DOI:** 10.21956/mep.19549.r28969

**Published:** 2019-05-12

**Authors:** Ken Masters

**Affiliations:** 1 Sultan Qaboos University

This review has been migrated. The reviewer awarded 4 stars out of 5 An interesting paper on the relationships between psychological needs satisfaction, motivation, and self-regulated learning strategies in medical residents. The authors begin by detailing the complexities involved in balancing the time and other demands of treating patients with staying abreast of medical knowledge through self-regulated learning. The authors also show the issues in relation the theoretical aspects of intrinsic motivation. The study has been well-conducted, using validated instruments, and the authors discuss their results in light of the literature.Within the limitations of self-reporting surveys, the researchers demonstrate a strong correlation between intrinsic motivation and self-regulated learning. A weakness of the paper is how the authors interpret this association. Logic may, perhaps, imply a one-way causative association, but the authors should be wary of implying this. Although they do not state so explicitly, their conclusion, discussing implications for medical education, implies this. It would have been better if the authors had explicitly stated that no causative association should be assumed.Still, the study itself is very valuable, and the paper is a worthwhile read.

Reviewer Expertise:

NA

No decision status is available

# Reviewer report: Reviewer response for version 1

**DOI:** 10.21956/mep.19549.r28967

**Published:** 2018-05-04

**Authors:** Subha Ramani

**Affiliations:** 1 Harvard Medical School, Brigham and Women's Hospital

This review has been migrated. The reviewer awarded 4 stars out of 5 The authors have conducted this study with rigor on an important topic. Motivation is something often talked about, but studies like this are needed to truly explore how it impacts learning and performance. Residency is a different stage of the learning cycle and results from studies on medical students cannot be automatically applied to residents' learning. for this reason, I compliment the authors for conducting this study.the paper is well written, well references, methodology shows rigor and appropriate for what they intended to study.I was surprised that autonomy and relatedness did not show a significant effect on the use of SRL strategies. In informal discussions teachers and residents tend to be focused on this issue. This study needs to taken to the next step using other methodology including qualitative methodology to explore this relationships between these SDT elements and performance.Motivation is a universal challenge and I believe all medical educators who teach trainees will find some aspect of this paper beneficial. Having said that, I concur with the other reviewer in hoping that this study is only a start.

Reviewer Expertise:

NA

No decision status is available

# Reviewer report: Reviewer response for version 1

**DOI:** 10.21956/mep.19549.r28968

**Published:** 2018-04-18

**Authors:** Adina Kalet

**Affiliations:** 1 New York University School of Medicine

This review has been migrated. The reviewer awarded 3 stars out of 5 This paper was an interesting read in that it is rigorous in measurement methodology and well written. There literature review is a good review of SDT and although limited in scope places the findings in context. The cross sectional study design is a major drawback in this domain of learning which is very dynamic and highly context and content dependent. Resident's feelings and reports on their own feeling of competence and use of strategies swings widely depending on what they are facing at the moment, how rested they are and is impacted highly by recent events. While I agree that the educational system should attend to providing trainees with a realistic and optimistic sense of their competence in order to motivate them, I am not certain what this study tells us about how learners become internally motivated and self-regulated, particularly at this late adolescence -early adult stage. Are these to be considered as traits or states and to what extent can the environment influence individual internalization of motivation? I assume this is the beginning of a research program aimed at exploring these issues in residency training and I look forward to more from this group.

Reviewer Expertise:

NA

No decision status is available
